# Fluoride release and uptake in enhanced bioactivity glass ionomer cement (“glass carbomer™”) compared with conventional and resin-modified glass ionomer cements

**DOI:** 10.1590/1678-7757-2018-0230

**Published:** 2019-02-21

**Authors:** Ammar M. H. R. HASAN, Sharanbir K. SIDHU, John W. NICHOLSON

**Affiliations:** 1 Queen Mary University of London , Barts & The London School of Medicine and Dentistry , Institute of Dentistry, Adult Oral Health , London , United Kingdom .; 2 Queen Mary University of London , Barts & The London School of Medicine and Dentistry , Institute of Dentistry, Dental Physical Sciences ; Bluefield Centre for Biomaterials, London , United Kingdom .

**Keywords:** Dental cements, Fluoride, Analysis

## Abstract

**Objectives:**

To study the fluoride uptake and release properties of glass carbomer dental cements and compare them with those of conventional and resin-modified glass ionomers.

**Materials and Methods:**

Three materials were used, as follows: glass carbomer (Glass Fill), conventional glass ionomer (Chemfil Rock) and resin-modified glass ionomer (Fuji II LC). For all materials, specimens (sets of six) were matured at room temperature for time intervals of 10 minutes, 1 hour and 6 weeks, then exposed to either deionized water or sodium fluoride solution (1000 ppm in fluoride) for 24 hours. Following this, all specimens were placed in deionized water for additional 24 hours and fluoride release was measured.

**Results:**

Storage in water led to increase in mass in all cases due to water uptake, with uptake varying with maturing time and material type. Storage in aqueous NaF led to variable results. Glass carbomer showed mass losses at all maturing times, whereas the conventional glass ionomer gained mass for some maturing times, and the resin-modified glass ionomer gained mass for all maturing times. All materials released fluoride into deionized water, with glass carbomer showing the highest release. For both types of glass ionomer, uptake of fluoride led to enhanced fluoride release into deionized water. In contrast, uptake by glass carbomer did not lead to increased fluoride release, although it was substantially higher than the uptake by both types of glass ionomer.

**Conclusions:**

Glass carbomer resembles glass ionomer cements in its fluoride uptake behavior but differs when considering that its fluoride uptake does not lead to increased fluoride release.

## Introduction

Glass ionomer cements are materials that have several applications in restorative dentistry, including functioning as liners and bases, full restoratives, pit-and-fissure sealants, and adhesives for the fixation of orthodontic brackets. ^1^ ^-^ ^3^

Bioactivity is an important feature of these materials, a phenomenon that has appeared in several observations. In saliva, glass ionomers have been shown to uptake calcium and phosphate ions with a resulting increase in hardness. ^4^ At the interface with the tooth, an ion-exchange process occurs over time that leads to the formation of a distinctive layer that provides a highly durable and strong bond between the cement and the tooth. ^5^ ^,^ ^6^ Lastly, at the bottom of pits and fissures, the morphology of the glass ionomer changes and a structure is formed, which is reported to be “enamel-like”. ^7^

These features have been exploited in a new type of glass ionomer material known as glass carbomer ^™^ . This material is formulated with hydroxyapatite as a secondary filler, ^8^ although previous reports suggested that the filler was fluorapatite. ^9^ ^,^ ^10^ However, solid-state NMR spectroscopy has showed that the filler is, in fact, hydroxyapatite. ^8^ Glass carbomer also contains a glass that has been washed with a strong mineral acid, such as hydrochloric acid. According to the description in the patent application, this is made so the surface layers of the glass are depleted in calcium, with most of the calcium ions lying within the core of the glass particles. ^11^ A final novel component of glass carbomer is a silicone oil consisting of linear polydimethylsiloxane molecules with functional hydroxyl groups. These hydroxyl groups can form hydrogen bonds with other cement components, thus preventing the silicone oil from leaching from the cement after being set. The precise function of silicone oil is not clear, although studies suggest the possibility that it is a toughening agent for what would otherwise be an extremely brittle material. ^12^

The fact that the glass is acid-washed, thus having reduced reactivity, in addition to the presence of the non-reactive hydroxyapatite filler, silicone oil makes the glass carbomer naturally slow to set. To overcome this, the recommendation is for these materials to be cured by application of heat from a dental curing light, applying heat for at least 20 seconds following placement. ^13^ ^,^ ^14^ This causes the glass carbomer to set to an acceptable extent relatively quickly, allowing the dentist to finish the process.

A particular feature of conventional glass ionomers is the release of fluoride. ^15^ ^-^ ^18^ This is considered a beneficial feature in general, ^19^ because it promotes the formation of fluorapatite at the tooth surface. This substance is slightly less soluble than hydroxyapatite, with a 10 ^-55.7^ solubility product at 25°C when compared to 10 ^-53.3^ for hydroxyapatite. ^20^ However, fluoride release levels are low and there is no clear evidence that such levels do actually confer any clinical benefit. ^21^ In addition to releasing fluoride, glass ionomers can uptake fluoride in conditions of high external fluoride concentration. ^15^ ^-^ ^17^

To date, there has been no scientific reports of the way glass carbomer behaves regarding fluoride uptake, although it has been reported to release fluoride ^22^ ^,^ ^23^ , and at higher levels than conventional glass ionomers. ^22^ This study aimed to compare the fluoride release of glass carbomer with a conventional and a resin-modified glass ionomers, and determining (a) whether glass carbomer can uptake fluoride and, if it does, (b) does such uptake result in increased fluoride release?

## Materials and methods

**Figure 1:**
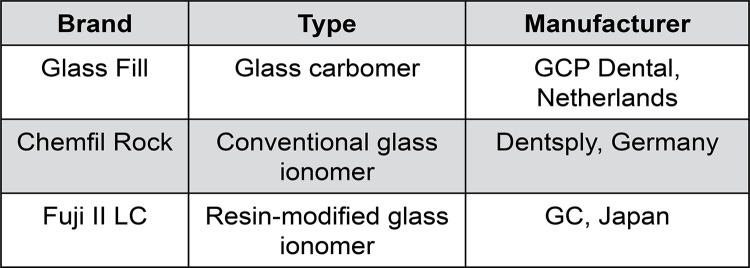
Materials employed

The materials used in these experiments are listed in Figure 1 , and comprised a glass carbomer, a conventional glass ionomer and a resin-modified glass ionomer. All materials are supplied in capsulated form. To prepare specimens, individual capsules were mixed in a dental rotary mixer (RotoMix, Espe, Seefeld, Germany), following, the freshly-mixed cement was extruded into a PTFE mold to prepare discs of dimensions 3 mm thickness x 5 mm diameter. Glass carbomer discs were heat-cured using a dental curing light (CarboLED, GCP Dental, Ridderkerk, The Netherlands) for 20 seconds on each side according to the manufacturer’s instructions, then matured in the mold for 10 minutes before being removed. Resin-modified glass-ionomer discs were light-cured using a dental curing light (CarboLED, GCP Dental, Ridderkerk, The Netherlands) for 20 seconds on each side according to the manufacturer’s instructions, then matured in the mold for 10 minutes before being removed. The conventional glass-ionomer was not treated in any way, but allowed to mature in the mold for 1 hour before being removed, according to the relevant ISO standard test. ^24^ Sets of six specimens for each combination of maturation time and storage medium were prepared.

Cement groups were prepared for testing in the following ways: one group was used immediately on removal from the mold, and two other groups were left to mature at room temperature for 1 hour or 6 weeks in plastic containers. After this, they were stored in either deionized water or sodium fluoride solution.

Two sets of specimens were prepared for each of the three maturation times. One set was initially weighed on a four-figure analytical balance, then placed into individual 5 cm ^3^ volumes of sodium fluoride (NaF) at 1000 ppm in fluoride (Fluoride standard, Sigma Aldrich, Dorset, UK), and left to mature for 24 hours, as previously described. ^25^ Following, the specimens were removed, dabbed dry with a tissue, weighed and had their fluoride concentration determined. The specimens were then transferred to individual 5 cm ^3^ volumes of deionized water and left to rest for additional 24 hours before being removed. The fluoride concentration in the storage solutions was then measured.

Mass changes were converted to percentage losses using the following equation:

% loss = mass change (mg)/original mass (mg) x 100%

Gains in mass were recorded as positive values; losses were recorded as negative values.

For each material, a second set of six specimens was stored in deionized water only. Each specimen was weighed and then placed in a 5 cm ^3^ volume of deionized water, removed after 24 hours, re-weighed and placed in a fresh 5 cm ^3^ volume of deionized water. The fluoride concentration in each volume of deionized water was measured at the end of the appropriate time period. Mass changes were calculated as previously explained.

Fluoride measurements were made with a fluoride ion-selective electrode (Elit 8221, NICO2000, London, UK) used in conjunction with a single-junction silver chloride electrode (Elit 001, NICO2000, London, UK), as previously described. ^25^ All samples were diluted 1:1 with TISAB IV solution (Sigma Aldrich, UK), which was added for complete decomplexation of the fluoride. Calibration plots were made on fluoride standards diluted 1:1 with TISAB IV, with calibration being conducted immediately prior to the measurement of experimental fluoride concentrations on all occasions.

Means and standard deviation values were calculated and differences were examined for significance by one-way ANOVA followed by Tukey’s HSD *post-hoc* test, considering a α=0.05 significance level.

## Results

Results are shown in Tables 1-3 . The first set of results ( Table 1 ) shows the mass changes for storage in sodium fluoride solution and deionized water, respectively, after the three different maturation times at room temperature. For all maturing periods, Glass Fill showed loss in NaF solution but gains in water, with variations affected by maturing. This consistent behavior was different from that shown by the two glass ionomers, where Chemfil Rock gained small amounts of mass in all cases, except in NaF solution after 6 weeks maturing. Fuji II LC showed similar results, with the one exception being a mass gain in NaF solution after the 1 hour maturing period.

**Table 1:** - Mass change on storage in water and NaF solution (groups that do not differ significantly are indicated with the same letters)

Brand	Ageing time	Storage medium	Mass change (%)	
Glass Fill	10 min	Water	18.2 (1.9)	A
		NaF solution	(-5.6) (4.5)	B
	1 h	Water	12.9 (1.4)	C
		NaF solution	(-4.0) (3.2)	B
	6 weeks	Water	10.0 (1.9)	D
		NaF solution	(-8.5) (8.2)	B
Chemfil Rock	10 min	Water	1.9 (0.6)	E
		NaF solution	2.7 (0.8)	E
	1 h	Water	5.2 (0.7)	F
		NaF solution	3.5 (1.3)	E
	6 weeks	Water	6.1 (0.6)	F
		NaF solution	(-4.0) (3.4)	B
Fuji II LC	10 min	Water	11.0 (1.3)	C
		NaF solution	11.3 (1.0)	C
	1 h	Water	5.2 (0.7)	F
		NaF solution	(-0.8) (1.3)	G
	6 weeks	Water	14.5 (1.4)	C C
		NaF solution	13.7 (1.3)	C

Table 2 shows the fluoride concentrations after 24 hours storage in solutions that were initially 1000 ppm in F ^-^ ion. In all materials matured for all three time periods, there was a reduction in fluoride concentration, showing the uptake of this ion. Glass Fill presented fluoride uptake values that significantly exceeded those of the two glass ionomers. Of the two glass ionomers, Fuji II LC presented uptake values to an extent that was also statistically significant. Regarding mass loss data, actual values depended on maturing periods prior to exposure to aqueous medium, but not in a way that was consistent with time. However, in all cases, maturation for 1 hour led to lowest fluoride uptake values.

**Table 2:** - Fluoride uptake by specimens (groups that do not differ significantly are indicated with the same letters)

Brand	Ageing time	Fluoride concentration, ppm	Fluoride uptake per specimen, mg	
Glass Fill	10 min	608.8 (40.0)	1.96 (0.20)	A
	1 h	751.1 (29.6)	1.24 (0.15)	B
	6 weeks	681.8 (29.8)	1.59 (0.15)	C
Chemfil Rock	10 min	861.6 (14.7)	0.69 (0.01)	D
	1 h	889.0 (8.7)	0.56 (0.04)	E
	6 weeks	794.2 (18.6)	1.03 (0.09)	B
Fuji II LC	10 min	926.1 (11.2)	0.37 (0.06)	E
	1 h	944.8 (7.7)	0.28 (0.04)	E
	6 weeks	920.4 (5.0)	0.40 (0.03)	E

Results in Table 3 show total fluoride release after 48 hours. For the specimens exposed to NaF solution, this represents the release after 24 hours in deionized water following the previous 24 hours being spent in the fluoride solution. For the specimens exposed to deionized water only, these results represent the sum of the release in the two 24-hour periods in fresh deionized water volumes. These results show that glass carbomer had the highest level of fluoride release but that there was no increase in fluoride release due to exposure to fluoride solution, a result that strongly contrasts with those for the two glass ionomers. Both Chemfil Rock and Fuji II LC showed a substantial and statistically significant increase in fluoride release after exposure to NaF solution for 24 hours.

**Table 3:** Fluoride retention (%) by specimens

Brand	Ageing time	Deionized water only	Exposed to NaF solution	Retention (%)
Glass Fill	10 min	39.2 (1.7)	36.2 (3.6)	100
	1 h	29.0 (0.6)	22.2 (2.7)	100
	6 weeks	19.3 (4.9)	18.2 (4.5)	100
Chemfil Rock	10 min	5.3 (1.0)	36.5 (1.8)	77.5
	1 h	3.8 (0.6)	24.6 (2.0)	81.3
	6 weeks	2.9 (1.3)	20.4 (5.9)	91.5
Fuji II LC	10 min	10.3 (1.1)	32.6 (2.8)	69.9
	1 h	10.0 (0.6)	32.4 (3.5)	59.4
	6 weeks	8.8 (1.1)	32.8 (3.3)	69.8

Table 3 includes results of retention percentage of fluoride. This was calculated by determining fluoride uptake values (shown in Table 2 ) and comparing it with the extra amount released following exposure to NaF solution. For example, Chemfil Rock maturated for 10 minutes presented 36.5 ppm fluoride release after exposure to NaF solution, which was 36.5 – 5.3 = 31.2 ppm more than for specimens exposed to deionized water. As shown in Table 3 , these specimens removed the equivalent of 138.4 ppm (i.e., 1000 – 861.6) from the NaF solution. Percentage release was thus given by:

% release = 31.2/138.4 x 100% = 22.5%

From this, it follows that retention was 77.5%.

The calculation for glass carbomer was complicated because specimens exposed to NaF solution subsequently released less fluoride than those exposed to deionized water. However, for specimens maturated for 10 minutes and 6 weeks this difference was not statistically significant, and in all cases the result can be simplified by recording the retention as 100%. This material was shown to uptake fluoride at all maturing times, and that increases in fluoride release do not occur, a finding that is consistent with 100% retention of absorbed fluoride.

## Discussion

The results obtained show that glass carbomer exhibits many similarities in its fluoride release and uptake behavior to conventional glass ionomer cements, and some important differences. The behavior in deionized water is very similar to that found for conventional glass ionomers, in that there is an increase in mass that can be attributed to the uptake of water. ^25^ Conventional glass ionomers are known to retain their dimensions rather than shrinking on setting, provided they are in an environment of at least 80% relative humidity, ^26^ and this feature has also been attributed to the readiness with which these materials uptake water.

By contrast, there was a distinct mass loss recorded for glass carbomer species exposed to sodium fluoride solution for 24 hours. Like the water uptake phenomenon, the extent of mass loss varied, non-linearly, with the degree of maturation. The comparable results for conventional glass ionomers are more varied, as shown in Table 1 . Previous studies have observed that such materials exposed to neutral fluoride aqueous solutions develop roughened surfaces, ^16^ a finding that has been attributed to an etching effect of neutral fluoride solutions on these surfaces. ^16^ Whether such etching effect occurs in the studied case is not clear; however, it would explain the observed mass loss. Further research is needed to determine whether glass carbomer materials are also etched in any way by aqueous fluoride solutions.

Like conventional glass ionomer cements, glass carbomer uptakes fluoride from these solutions. Usually, this has been observed indirectly from the observation that fluoride release by glass ionomers is increased by prior exposure to aqueous fluoride solutions ^27^ ^-^ ^29^ and, in the case of experimental fluoride-free glass ionomers, fluoride release can be introduced as a result of exposure to such fluoride solutions. ^30^

Despite this uptake, and unlike our findings for the two glass ionomers, no increase in fluoride release by the glass carbomer was found. This result shows that fluoride becomes incorporated irreversibly in glass carbomer, suggesting a different type of binding than the one that occurs in glass ionomer cements. This may be the result of the presence of hydroxyapatite in the formulation. Hydroxyapatite is known to irreversibly uptake fluoride to a level equivalent to approximately three atomic layers depth. ^31^ This layer is of much lower solubility than pure hydroxyapatite and as a result, it causes the fluoride to be strongly retained within the material.

For both fluoride release and uptake, differences were observed between specimens matured for different periods of time. Glass ionomers undergo maturation processes that are imperfectly understood, but that result in improvements in strength and translucency. ^1^ ^,^ ^12^ These changes are also associated with increases in the proportion of strongly bound water within the cement. Previous studies on fluoride uptake by glass ionomer cements have reported variations depending on the extent of maturation, ^25^ a result confirmed in this study. The results obtained show that the link between fluoride uptake and degree of maturation for conventional glass ionomer cements is also valid for the glass carbomer.

This study was entirely undertaken at room temperature (20-22°C). In clinical service, the studied materials are employed at body temperature, i.e., 37°C. However, it is unlikely that the phenomena we have observed are restricted to lower temperatures. Therefore, we consider that our findings are relevant to the clinical use of these materials, and that glass carbomer probably does not release the fluoride taken up when used at body temperature.

## Conclusions

Glass carbomer was shown to have differences and similarities with conventional glass ionomer cements. In water, it shows a degree of mass gain, as the result of water uptake. By contrast, in sodium fluoride solution, it presented mass loss, which may be attributed to the etching effect of this solution. The etching effect is much more pronounced in glass carbomer than in conventional glass ionomers exposed to sodium fluoride solutions.

Fluoride release by glass carbomer was shown to vary depending on the maturing period, in which is similar to conventional glass ionomer cements. In all cases, there was a steady decrease in fluoride release as maturing time increased, a result that can be attributed to the changes known to occur in these materials as they mature. However, the exact cause of the decline is not known.

Finally, glass carbomer has been shown to uptake fluoride from sodium fluoride solutions, which is also similar to glass-ionomers. Fluoride uptake by the material was substantially higher than both glass ionomers, however, despite this finding, there was no increase in fluoride release levels subsequently observed. This result strongly contrasts the behavior of glass ionomers cements, and can be attributed to the presence of hydroxyapatite as a secondary filler in glass carbomer, since this substance is known to irreversibly uptake fluoride.
